# Deletion of Ovarian Hormones Induces a Sickness Behavior in Rats Comparable to the Effect of Lipopolysaccharide

**DOI:** 10.1155/2015/627642

**Published:** 2015-01-29

**Authors:** Hamid Azizi-Malekabadi, Mahmoud Hosseini, Masoume Pourganji, Hoda Zabihi, Mohsen Saeedjalali, Akbar Anaeigoudari

**Affiliations:** ^1^Department of Biology, Faculty of Basic Sciences, Islamic Azad University, Isfahan (Khorasgan) Branch, Isfahan, Iran; ^2^Neurocognitive Research Center and Department of Physiology, School of Medicine, Mashhad University of Medical Sciences, Azadi Square, Mashhad 9177947564, Iran; ^3^Neurogenic Inflammation Research Center and Department of Physiology, School of Medicine, Mashhad University of Medical Sciences, Mashhad, Iran; ^4^Pharmacological Research Center of Medicinal Plants, School of Medicine, Mashhad University of Medical Sciences, Mashhad, Iran; ^5^Mashhad Technical Faculty, Technical and Vocational University, Mashhad, Iran

## Abstract

Neuroimmune factors have been proposed as the contributors to the pathogenesis of sickness behaviors. The effects of female gonadal hormones on both neuroinflammation and depression have also been well considered. In the present study, the capability of deletion of ovarian hormones to induce sickness-like behaviors in rats was compared with the effect lipopolysaccharide (LPS). The groups were including Sham, OVX, Sham-LPS, and OVX-LPS. The Sham-LPS and OVX-LPS groups were treated with LPS (250 *μ*g/kg) two hours before conducting the behavioral tests. In the forced swimming (FST), the immobility times in both OVX and Sham-LPS groups were higher than that of Sham (*P* < 0.001). In open-field (OP) test, the central crossing number by OVX and Sham-LPS groups were lower than Sham (*P* < 0.001) while there were no significant differences between OVX-LPS and OVX groups. In elevated plus maze (EPM), the percent of entries to the open arm by both OVX and Sham-LPS groups was lower than that of Sham group (*P* < 0.001). The results of present study showed that deletion of ovarian hormones induced sickness behaviors in rats which were comparable to the effects of LPS. Moreover, further investigations are required in order to better understand the mechanism(s) involved.

## 1. Introduction

Sickness behavior is a behavioral pattern that occurs following the infections and tissue injury in many mammalian species [1]. The behavioral features are including malaise, hyperalgesia, pyrexia, disinterest in social interactions, lethargy, behavioral inhibition, reduced locomotor activity, lower exploration and grooming behaviors, reduction of reproductive performance, anhedonia, somnolence and sleepiness, anorexia and weight loss, failure to concentrate, and anxiety [1]. Proinflammatory cytokines, such as interleukin-1 (IL-1), tumor necrosis factor (TNF-*α*), and interleukin-6 (IL-6), can induce sickness behavior [2]. Proinflammatory cytokines can block the energy consuming processes including locomotor, neurocognitive, and reproductive activities [3]. Depression, the second most common chronic disease, is expanding in the world while about half of the patients with depression are unaware of their disease or their disease are diagnosed else [4]. Depression occurs in children, adolescents, adults, and the elderly as a result of the combination of states of sadness, loneliness, irritability, absurdity, despair, confusion, and shame and the physical symptoms, for example, reduction of locomotor activity [4]. In addition, depressed patients show the symptoms such as, anorexia, weight loss, fatigue, lethargy, and hyperalgesia [1]. Depression is suggested to be an immunoinflammatory disorder and neuroimmune factors have been proposed as the contributors to its pathogenesis [5, 6]. Increased levels of the inflammatory markers such as C-reactive protein (CRP), haptoglobin, IL-6, and TNF-*α* have been reported to be associated with depression [7, 8]. Thus, there are striking behavioral and inflammatory similarities between sickness behavior and clinical depression.

Ovarian hormones have been well known to have an important role in the regulation of mood and cognitive functions [9]. They also influence synthesis, release, reuptake, enzymatic inactivation, and number of receptors for many neurotransmitters such as serotonin, dopamine, and norepinephrine [9]. There is also evidence that ovarian hormones such as estrogen influence on the production and bioactivity of proinflammatory cytokines, such as TNF-*α*, IL-1, and IL-6 [10]. It has been reported that a reduced level of ovarian hormones in menopausal women and ovariectomized rats causes to increase the proinflammatory cytokines production [9]. It has also been shown that estrogen reduces TNF-*α* in glial cells in response to endotoxins [11]. A part of the effects of estrogen on cerebral blood supply has been attributed to its anti-inflammatory effects [9]. It has been reported that the brain atrophy occurs particularly in the hippocampus and parietal lobe and the risk for depression increases as the level of estrogen is declined in the perimenopausal period [12–14].

Lipopolysaccharide (LPS), an endotoxin of gram-negative bacteria, is used for inducing chronic inflammation in the rodent [15]. LPS-induced activation of peripheral innate immune cells elicits secretion of inflammatory cytokines, including IL-1, IL-6, and TNF-*α*. Systemic injection of LPS causes spectrum behavior responses such as sickness behavior that animals show reduction in locomotor activity, exploration and feeding [15]. It also has been reported that peripheral and central administration of LPS increases the body temperature, disturbs sleep, suppresses food intake and body weight, and finally induces a depression-like behavior in rabbits, rats, and mice [16–18]. Regarding the facts that inflammation induces a sickness like behavior and regarding the role of ovarian hormones on both inflammation responses and regulation of the CNS functions, the present study aimed to evaluate the effect of deletion of ovarian hormones and LPS on sickness behaviors in rats.

## 2. Materials and Methods

### 2.1. Animals and Drugs

Sixty female Wistar rats, 12 weeks old (240 ± 10 g), were used. The animals were housed in 4-5 per standard cages, at room temperature (22 ± 2°C) on a 12 h light/dark cycle. Food and water were available* ad libitum* properly. Animal handling and all related procedures were approved by the Mashhad Medical University Committee on Animal Research. The animals were divided into four groups: (1) Sham (*n* = 20), (2) OVX (*n* = 10), (3) Sham-lipopolysaccharide (Sham-LPS; *n* = 20), and (4) ovariectomized-lipopolysaccharide (OVX-LPS; *n* = 10). In Sham and Sham-LPS groups, 9-10 animals which had proestrous phase were selected and used for the behavioral studies.

The animals in the Sham-LPS and OVX-LPS groups were treated by single injections of LPS (250 *μ*g/kg; i.p.) before behavioral tests. The animals of Sham and OVX groups received 1 mL/kg of saline instead of LPS. Ketamine was purchased from Alfasan Company (The Netherlands). LPS was purchased from sigma (Sigma Chemical Co.).

### 2.2. Surgery

Before the surgery, the rats were permitted 15 days for acclimatization to the animal house. The animals were ovariectomized under ketamine (100 mg/kg, i.p.) anesthesia. Anesthesia was confirmed by reduced respiratory rate and no response to gentle pinching of foot pad. Abdominal incision was made through the skin of the flank of the rats and ovaries and ovarian fats were removed. Ovaries were isolated by ligation of the most proximal portion of the oviduct before removal. The same procedure was performed on the sham rats except that the wound was closed without removing the ovaries [19]. The animals were allowed 8 weeks to recover from the surgeries and to diminish the level of ovarian hormones.

### 2.3. Vaginal Cytology

It was carried out in Sham and Sham-LPS groups to select the animals with proestrous stage for behavioral studies. The female rat estrous cycle is 4-5 days and includes 4 phases: (1) proestrous stage where estrogen levels are very high and its typical cell pattern is smears including primarily epithelial cells with large nuclei. (2) Estrous stage, typical cell pattern for this order, is smears containing primarily cornified epithelial cells. (3) Metaestrous stage comprises cornified cells, primarily cornified cells, and sometimes a few epithelial cells. (4) Diestrous with typical cell pattern consists of large numbers of leukocytes with scattered nucleated epithelial and cornified cells in smears. To ensure that the female rats were cycling, vaginal cytologies were started 1 week before each experience and continued every day. Rats were held in a nonstress position and quickly lavaged with approximately 1 mL of saline. Slides were read using light microscopy, and estrous categories were classified based on cytological characteristics [20, 21].

### 2.4. Behavioral Procedures

#### 2.4.1. Forced Swimming Test

All animals were compromised to the test room environment for 1 h before the beginning of the experiment. During the forced swimming test (FST), the animals were placed in a glass cylindrical tank with 60 cm height and 38 cm width which was filled with water (24 ± 1°C) to the depth of 40 cm. The water was changed between each animal. In the first day, the rats were placed inside the water cylinder for 15 min (pretest) and then the animals were placed individually inside the water cylinder for 5 min for the following three alternative days (test days) [22, 23]. Two hours before each test, the rats were injected with either LPS 250 *μ*g/kg (Sham-LPS and OVX-LPS groups) or saline (Sham- and OVX groups) [21, 23]. The time of floating (immobility) during the FST was recorded for 5 min. Rats were considered immobile when they floated in the water; they only performed movements that enabled them to keep their head above the water. After the FST, the rats were dried in a heated cage and then they were returned to their home cages.

#### 2.4.2. Open-Field Test

Open-field test was carried out for studying the depression-like behaviors of the animals. In the present study, the open-field measurement was done by a Plexiglass apparatus with an area of 100 × 100 cm and height of 40 cm. The inside of the apparatus was divided into 16 equal squares using a black line. In addition, within the apparatus was divided to two zones called peripheral and central zones [22, 23]. All the rats were familiarized with the test environment by being placed in the room for 1 h before beginning the experiment. During the experiment, a low-level light was used to reduce anxiety (20 lux). Each animal was placed in the central zone and its movement was recorded by a digital camera for 5 min [24], and the following criteria were calculated: (1) the crossing number in the central zone, (2) the crossing number in the peripheral zone, (3) the traveled distance in central zone, (4) the traveled distance in peripheral zone, (5) the time spent in central zone, (6) the time spent in in peripheral zone, (7) the total crossing number, and (8) the total traveled distance. Two hours before each test, the rats were injected with either LPS 250 *μ*g/kg (Sham-LPS and OVX-LPS groups) or saline (Sham- and OVX groups) [22, 23].

#### 2.4.3. Elevated Plus Maze

The elevated plus maze (EPM) was made of 4 arms (50 cm length × 10 cm width) elevated 100 cm above the floor. Two of the arms which are named closed arms had 40 cm high dark walls and the other two which are named open arms had 0.5 cm high ledges [25]. The between angle of arms was 90°. The EPM was placed in a quit dimmed in order to provide 10–20 lux of illumination on the open arms and <0.5 lux within closed arm. In the test day the rats were placed in the center of the apparatus facing a closed arm then returned to the home cage after 5 min. After each session, the apparatus was cleaned. In this behavioral test, percent of time spent in the open and closed arms and the number of entries in the closed and closed arms were measured [26].

### 2.5. Statistical Analysis

The data were expressed as mean ± SEM. Repeated measures ANOVA were run followed by Tukey post hoc comparisons test. The criterion for the statistical significance was *P* < 0.05.

## 3. Results

### 3.1. Forced Swimming Test

**Figure 1:**
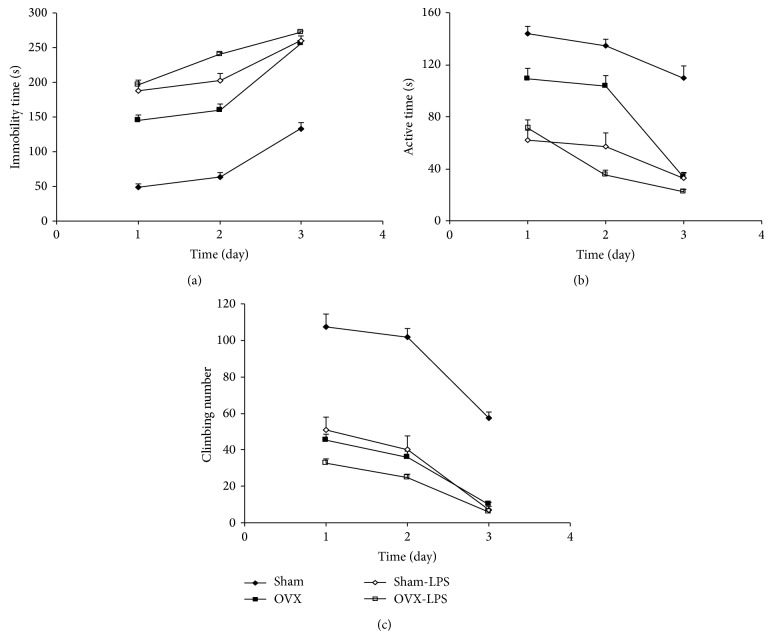
Comparison of immobility times (a), active times (b), and the number of climbing (c) in the forced swimming test between four groups. Data are expressed as mean ± SEM (*n* = 10 in each group). The repeated measures ANOVA showed that the immobility times in the OVX group were higher while, the active times were lower than those of the Sham group (*P* < 0.001). The immobility times in the Sham-LPS group were higher while, the active times than those of the Sham group (*P* < 0.001). In OVX-LPS group, the immobility times were higher while the active times were lower than those of the OVX group (*P* < 0.001). The climbing number in both Sham-LPS and OVX groups were lower than that of the Sham group (*P* < 0.001). There were no significant differences between OVX-LPS and OVX group.

The immobility times in both the OVX and Sham-LPS groups were higher than that of the Sham group (*P* < 0.001; Figure 1(a)). The immobility times in both the Sham-LPS and OVX-LPS groups were also higher than that of and OVX group (*P* < 0.01–*P* < 0.001; Figure 1(a)).

The results of FST also showed that the active times in both the OVX and Sham-LPS groups were lower than that of the Sham group (*P* < 0.001; Figure 1(b)). The active times in the animals of both Sham-LPS and OVX-LPS groups were lower than that of OVX group (*P* < 0.001; Figure 1(b)).

The climbing number in the OVX and Sham-LPS groups was lower than that of the Sham group (*P* < 0.001; Figure 1(c)); however, there was no significant difference between the Sham-LPS and OVX groups. There were no significant differences between OVX-LPS and OVX groups when the climbing number was compared (Figure 1(c)).

### 3.2. Open-Field Test

**Figure 2:**
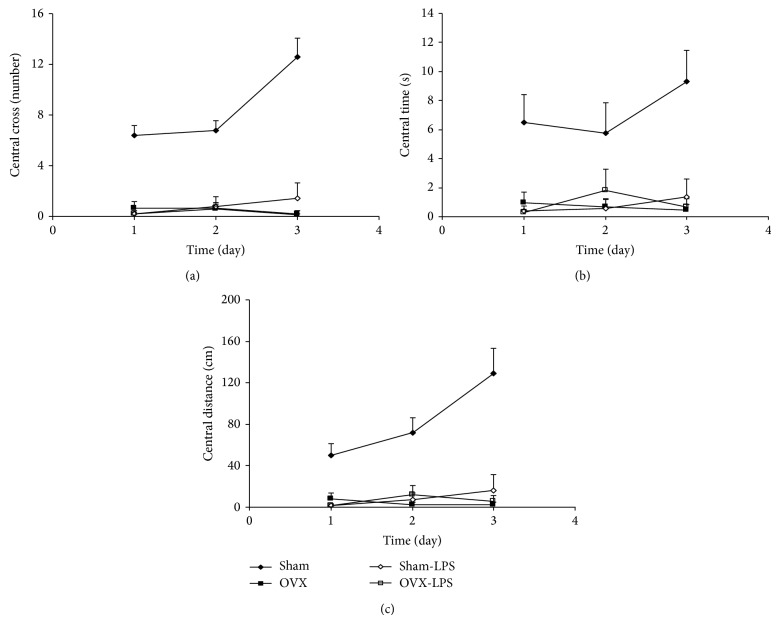
Comparison of central crossing number (a), the time spent in central zone (b), and the traveled distance in central zone (c) in the open field test between four groups. Data are expressed as mean ± SEM (*n* = 10 in each group). The time spent and the traveled distance in the central zone in both OVX and Sham-LPS groups were lower than that of the Sham group (*P* < 0.001). There were no significant differences between OVX-LPS and OVX groups.

The results of the open-field test showed that the number of crossing in the central zone in the OVX group was lower than that of the Sham group (*P* < 0.001). As shown in Figure 2(a), the crossing number in the central zone in Sham-LPS group was significantly lower than that in the Sham group (*P* < 0.001). There were no significant differences between Sham-LPS and OVX group. The results also showed that the central zone crossed number by the animals of OVX-LPS group was not different form that of OVX group (Figure 2(a)). As Figures 2(b) and 2(c) show the time spent and the traveled distance in central zone by the animals of OVX group were lower than that of Sham group (*P* < 0.001). The animals of Sham-LPS group spent lower times and traveled lower distance in central zone compare to Sham group (*P* < 0.001; Figures 2(b) and 2(c)). There were no significant differences between Sham-LPS and OVX groups when the time spent and the traveled distance in the central zone were compared (Figures 2(b) and 2(c)). There were also no significant differences between OVX-LPS and OVX group neither in time nor in traveled distance in central zone (Figures 2(b) and 2(c)).

**Figure 3:**
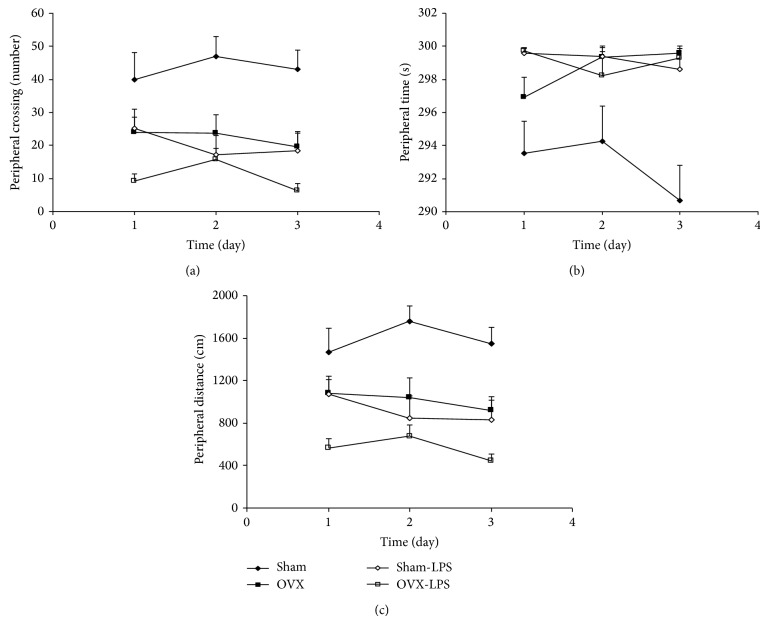
Comparison of peripheral crossing number (a), the time spent in peripheral zone (b), and the traveled distance in peripheral zone (c) in the open field test between four groups. Data are expressed as mean ± SEM (*n* = 10 in each group). The number of crossing, the time spent, and the traveled distance in the peripheral zone in the OVX and Sham-LPS group groups were lower than that of the Sham group (*P* < 0.01–*P* < 0.001). The peripheral crossing number by the animals of OVX-LPS group was not different than that of OVX group. There was also no significant differences between OVX-LPS and OVX group in the time spent in peripheral zone and peripheral crossing number; however, the traveled distance in the peripheral zone by the animals of OVX-LPS group was lower than that of OVX group (*P* < 0.05).

The results of the open-field test also showed that the number of crossing in the peripheral zone in the OVX group was lower than that of the Sham group (*P* < 0.01; Figure 3(a)). As shown in Figure 3(a), the crossing number in the peripheral zone in Sham-LPS group was significantly lower than that in the Sham group (*P* < 0.01); however, there were no significant differences between Sham-LPS and OVX group (Figure 3(a)). The peripheral crossing number by the animals of OVX-LPS group was not different than that of OVX group (Figure 3(a)). As Figures 3(b) and 3(c) show, the time spent and the traveled distance in peripheral zone by the animals of OVX group were lower than that of Sham group (*P* < 0.01–*P* < 0.001). The animals of Sham-LPS group spent lower times and traveled distance in the peripheral zone compare to Sham group (*P* < 0.001; Figures 3(b) and 3(c)). There were no significant differences between Sham-LPS and OVX groups when the time spent and the traveled distance in peripheral zone were compared (Figures 3(b) and 3(c)). There were also no significant differences between OVX-LPS and OVX groups in the time spent in the peripheral zone; however, the traveled distance in the peripheral zone by the animals of OVX-LPS group was lower than that of OVX group (*P* < 0.05; Figures 3(b) and 3(c)).

**Figure 4:**
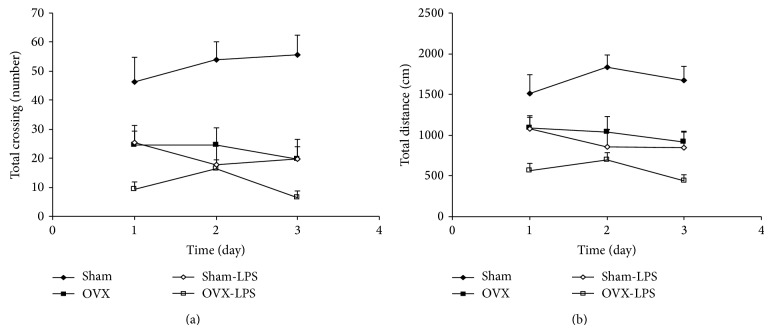
Comparison of total crossing number (a) and the total traveled distance (b) in the open field test between four groups. Data are expressed as mean ± SEM (*n* = 10 in each group). The total number of crossing and the total traveled distance in the OVX and Sham-LPS groups were lower than that of the Sham group (*P* < 0.01–*P* < 0.001). The total crossing number and the total traveled distance by the animals of OVX-LPS group also was lower than that of OVX group (*P* < 0.05).

The results of the open-field test also showed that the total number of crossing in the OVX group was lower than that of the Sham group (*P* < 0.001). As shown in Figure 4(a), the total crossing number in Sham-LPS group was significantly lower than that in the Sham group (*P* < 0.001); however, there were no significant differences between Sham-LPS and OVX group. The total crossing number by the animals of OVX-LPS group also was lower than that of OVX group (*P* < 0.05; Figure 4(a)). As Figure 4(b) shows, the total traveled distance by the animals of OVX group was lower than that of Sham group (*P* < 0.01). The animals of Sham-LPS group traveled shorter distance compared to Sham group (*P* < 0.001; Figure 4(b)). There were no significant differences between Sham-LPS and OVX groups when the traveled distance was compared. The total traveled distance by the animals of OVX-LPS group was lower than that of OVX group (*P* < 0.05; Figure 4(b)).

### 3.3. Elevated Plus Maze

**Figure 5:**
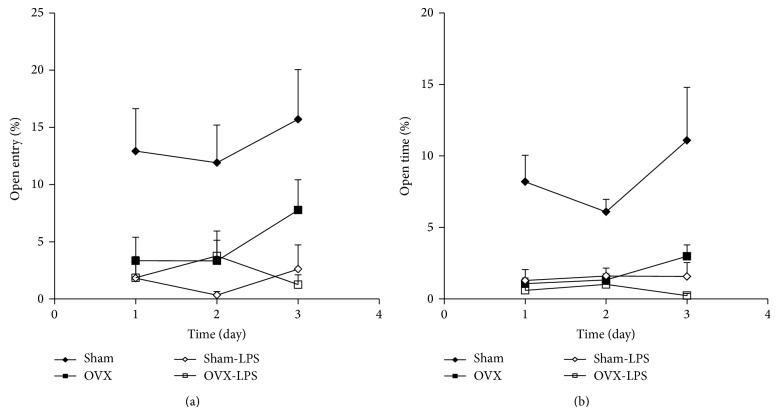
Comparison of the percent number of entries to the open arm (a) and the percent of time spent in open arm (b) in the elevated plus maze test between four groups. Data are expressed as mean ± SEM (*n* = 10 in each group). The percent number of entries into and the percent of time spent in the open arm by the animals of OVX and Sham-LPS groups were lower than that of Sham group (*P* < 0.01–*P* < 0.001). There were no significant differences between OVX-LPS and OVX groups.

In elevated plus maze, the percent of entries to the open arm by the animals of OVX group was lower than that of Sham group (*P* < 0.01; Figure 5(a)). In Sham-LPS group also, the percent of entries to the open arm was lower than that of Sham group (*P* < 0.001; Figure 5(a)). There were no significant differences between Sham-LPS and OVX groups and also there were no significant differences between OVX-LPS and OVX groups when the percent of entries to the open arm was compared between these two groups (Figure 5(a)). The results also showed that the percent of time spent in open arm by the animals of OVX group was lower than that of sham group (*P* < 0.001; Figure 5(b)). The animals of Sham-LPS group also spent lower times in the open arm compared to Sham group (*P* < 0.001; Figure 5(b)). There were no significant differences between Sham-LPS and OVX group when the percent of time spent in the open arm was compared (Figure 5(b)). There were also no significant differences between OVX-LPS and OVX groups in the time spent in the open arm (Figure 5(b)).

**Figure 6:**
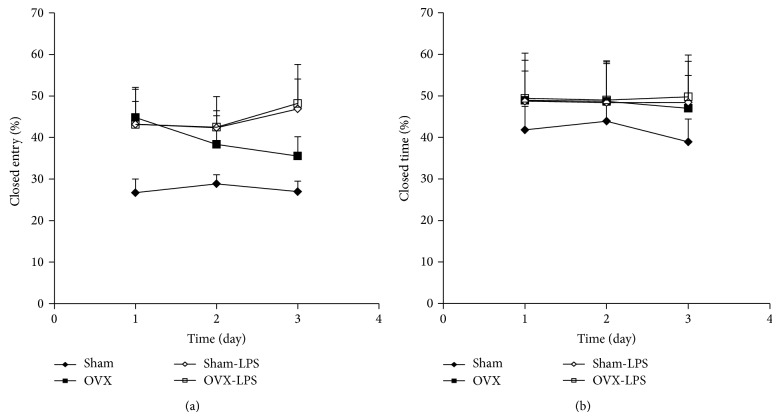
Comparison of the percent number of entries to the closed arm (a) and the percent of time spent in closed arm (b) in the elevated plus maze test between four groups. Data are expressed as mean ± SEM (*n* = 10 in each group). There were no significant differences between Sham and OVX groups in the percent of entries to the closed arm. The percent of entries to the closed arm by the animals of Sham-LPS group was higher than that of Sham groups (*P* < 0.01). There were no significant differences between four groups in the percent of time spent in the closed arm.

There were no significant differences between Sham and OVX groups in the percent of entries to the closed arm (Figure 6(a)). The percent of entries to the closed arm by the animals of Sham-LPS group was higher than that of Sham groups (*P* < 0.01; Figure 6(a)). The results showed no significant differences between OVX-LPS and OVX groups (Figure 6(a)). There were no significant differences between Sham and OVX groups in the percent of time spent in the closed arm (Figure 6(b)). There were no significant differences between Sham-LPS and Sham groups in the percent of time spent in the closed arm (Figure 6(b)). The results showed no significant differences between OVX-LPS and OVX groups (Figure 6(b)).

## 4. Discussion

It has been well documented that systemic microbial infectious activates immune system which the later triggers a set of behavior changes called sickness behavior [17]. Both systemic and central administrations of LPS activate proinflammatory cytokines in several areas of the brain including the hippocampus, hypothalamus, and diencephalic structures in rodents which is accompanied with a complex of behavioral comparable to the cytokine-induced sickness and depression-like behaviors [27–30]. In the present study, the corresponding depressive symptoms and sickness behaviors were observed in rats treated by LPS. The results of the present study showed that immobility times in the LPS group were higher than those of the control group in the forced swimming test. Conversely, the activity time and the climbing were decreased by LPS. It seems that peripheral administration of a medium dose of LPS induces depressive like behaviors. It is likely that the injection of LPS in 3 times which was carried out in the present study is resulted in a cumulative response, because it has been previously reported that the effect of LPS may be seen even 28 days after injection [31].

It has been previously reported that administration of LPS in doses lower than the doses used in the present study resulted in avoidance of the central area of the open-field by the rats [15, 31]. The results of present study also showed that the crossing number in the central area of the open-field by the animals of the LPS-treated groups was lower than that of the control ones which may be another evidence for sickness and depression like behaviors due to administration of LPS. In consistency with the results of present study, a large number of evidences has confirmed the role of inflammation in depression. For example, treatment with interleukin-2 (IL-2) or interferon-*γ* (IFN)-*γ* in patients with cancer is accompanied with depressive symptoms [32, 33]. On the other hand, depressed patients exhibit all the cardinal features of inflammation [32]. It has also been suggested that various anti-inflammatory manipulations have antidepressant effects in experimental animals and humans [34]. For example, IL-6 knockout mice had a reduced depressive-like behavior in the forced swim, tail suspension, learned helplessness, and sucrose preference tests [35]. Knockout of the IL-1 receptor and administration of IL-1 receptor antagonist also blocked stress-induced depressive-like behavior in the sucrose preference, social exploration tests, escape deficits, anhedonia, and reduces social behavior [36]. It has been suggested that LPS activates norepinephrine and serotonin metabolism in the brain [37] which are essential to the regulation of emotion and psychomotor functions [38, 39]. The effects of LPS on depression and sickness behaviors which were seen in the present study might be elucidated by altered concentrations of these neurotransmitters in the brain.

It has also been reported that LPS activates the hypothalamic-pituitary-adrenal axis thus increasing adrenocorticotropic hormone (ACTH) and glucocorticoids [15]. In addition, LPS stimulates sympathetic system activity [40] that these effects are similar to what take place during anxiety. It has been previously reported that injection of 3 *μ*g/kg and 10 *μ*g/kg LPS to substantia nigra induced an anxiety-like behavior in rats which was presented by decreasing in the percentage of the time spent in the open arms, the number of open-arm entries and the number of crossing in elevated plus maze [41]. In the present study, administration of LPS decreased the percent number of entries into as well as the percent time spent in open arms, however, there were no significant differences in the number entries into and the time spent in the closed arms.

Ovarian hormones not only play an important role in reproductive behavior but are also involved in emotion, memory, neuronal survival, and the perception of somatosensory stimuli [42, 43]. Researchers have shown that mood disorders in women are more common than men [44]. This fact has been attributed to hormonal changes in premenstrual and postpartum periods which has also been confirmed in hypoestrogenic conditions occurred during medical surgery or menopause [45]. It has also been shown that deletion of ovarian hormones induces a depressive-like behavior in rats [46, 47]. Using forced swimming and tail suspension tests, an increase in immobility time and a decrease in active behaviors were reported 2–4 weeks after ovariectomy in both rats and mice which were partially reduced by estradiol injection [43, 48]. In the present study, the ovariectomized rats showed higher immobility and lower active times compared to sham operated ones when the animals were examined in FST which was comparable to the effects of LPS.

The results of present study also showed exploratory behaviors in open-field test were decreased in both OVX and Sham-LPS groups compared to the Sham group. These data confirm the similar capability of ovarian hormones deletion and inflammation induced by LPS to induce sickness behaviors and depression.

A large number of evidence show that LPS [15, 37] and ovarian hormonal [9] influence concentration of many neurotransmitters in the brain. It was shown that injection of estradiol lead to increase serotonin uptake in frontal cortex and hypothalamus in ovariectomized rats [9, 49]. Researchers have also shown that estrogen competes with tryptophan, the precursor of serotonin, for binding sites on plasma albumin which may lead to an increased availability of this amino acid to the CNS [9]. In addition estrogen reduces mono amino oxidase (MAO) activity [50] and decreases the expression of catechol-O-methyltransferase (COMT) thus increasing the levels of catecholamines and serotonin in the brain [9] while LPS increases destruction of these neurotransmitters [51]. Estrogen has also been shown to have a neuroprotective effect on dopaminergic neurons [52, 53] and prevent degeneration of neurons [54]. All of these data confirm that ovarian hormones elimination induces a sickness behavior in rats.

It has been reported that anxiety-like behavior occurred in OVX rats when the animals were examined in the elevated plus maze test [55]. Clinical studies also show that anxiety disorders occur in 50% of menopausal women [56]. Similarly, LPS injection in mice and rats was also followed by an anxiety-like behavior in elevated plus maze [57]. The results of present study also showed that the percent number of entries into as well as the percent of the time spent in open arm of elevated plus maze decreased after both ovariectomy and LPS injection.

EPM is a well-known test which has been frequently used for evaluation of anxiety like behaviors in which the percent of the time in open arms is compared between the groups [15, 41, 55]. Some of researchers believe that evaluation of the open and closed arms entries and the time spent in each part of the maze are not enough to judge about the anxiety in animals and other variables such as head-dipping, rearing, and especially grooming are needed to be analyzed [57–59]. In the present study anxiety and depression like behaviors due to deletion of ovarian hormones as well as injection of LPS were confirmed using EPM, OP, and FST tests. It has previously reported that a FST test has an anxiogenic effect in the rodents if it is done before the EPM test [60]. Regarding this idea, the order of the tests were OP, EPM, and FST which were done in consecutive weeks. As it is seen in the Figure 5(a), the percent of time spent in open arms by the animals of Sham group was increased in the third trial compared to the first and the second trials. In consistency with these results, it has been previously suggested that the animals show an anxiety like behavior during the first trial due to a fear of heights which is reduced during the latter trials [61]. This phenomenon which has also been reported by examination of the anxiolytic effects of the drugs such as benzodiazepines was considered as “one-trial tolerance” in EPM [60, 62–64]. Finally it has been previously reported that exposure to EPM affects the expression of an immediate early gene c-Fos in the limbic brain regions [65–67]. Stronger and more widespread c-Fos induction has also been reported due to stress conditions [65, 68, 69] which is suppressed by the anxiolytic drugs such as benzodiazepines [70]. Regarding these facts, the experiments with c-Fos are suggested to be done in the future to better understand the correlation between LPS treatment and ovariectomy to induce anxiety and sickness behaviors.

In conclusion, the results of present study using forced swimming, open field, and elevated plus maze tests showed that deletion of ovarian hormones induced anxiety and depression like behaviors which might be considered as a sickness behavior in rats. These behaviors were comparable to the effects of lipopolysaccharide. Therefore, some common mechanism(s) might be suggested; however, it needs to be more investigated in the future.

## References

[1] Maes M., Berk M., Goehler L. (2012). Depression and sickness behavior are Janus-faced responses to shared inflammatory pathways. *BMC Medicine*.

[2] Burton M. D., Sparkman N. L., Johnson R. W. (2011). Inhibition of interleukin-6 trans-signaling in the brain facilitates recovery from lipopolysaccharide-induced sickness behavior. *Journal of Neuroinflammation*.

[3] Maes M., Kubera M., Obuchowiczwa E., Goehler L., Brzeszcz J. (2011). Depression's multiple comorbidities explained by (neuro)inflammatory and oxidative & nitrosative stress pathways. *Neuroendocrinology Letters*.

[4] Sharp L. K., Lipsky M. S. (2002). Screening for depression across the lifespan: a review of measures for use in primary care settings. *American Family Physician*.

[5] Dantzer R., O'Connor J. C., Lawson M. A., Kelley K. W. (2011). Inflammation-associated depression: from serotonin to kynurenine. *Psychoneuroendocrinology*.

[6] Maes M., Yirmyia R., Noraberg J. (2009). The inflammatory & neurodegenerative (I&ND) hypothesis of depression: leads for future research and new drug developments in depression. *Metabolic Brain Disease*.

[7] Dowlati Y., Herrmann N., Swardfager W. (2010). A meta-analysis of cytokines in major depression. *Biological Psychiatry*.

[8] Howren M. B., Lamkin D. M., Suls J. (2009). Associations of depression with c-reactive protein, IL-1, and IL-6: a meta-analysis. *Psychosomatic Medicine*.

[9] Shepherd J. E. (2001). Effects of estrogen on congnition mood, and degenerative brain diseases. *Journal of the American Pharmaceutical Association*.

[10] Nordell V. L., Scarborough M. M., Buchanan A. K., Sohrabji F. (2003). Differential effects of estrogen in the injured forebrain of young adult and reproductive senescent animals. *Neurobiology of Aging*.

[11] Członkowska A., Ciesielska A., Gromadzka G., Kurkowska-Jastrzebska I. (2006). Gender differences in neurological disease: role of estrogens and cytokines. *Endocrine*.

[12] Murphy D. G. M., DeCarli C., McIntosh A. R. (1996). Sex differences in human brain morphometry and metabolism: an in vivo quantitative magnetic resonance imaging and positron emission tomography study on the effect of aging. *Archives of General Psychiatry*.

[13] Ferrini R. L., Barrett-Connor E. (1998). Sex hormones and age: a cross-sectional study of testosterone and estradiol and their bioavailable fractions in community-dwelling men. *The American Journal of Epidemiology*.

[14] Khastgir G., Studd J. (1998). Hysterectomy, ovarian failure, and depression. *Menopause*.

[15] Swiergiel A. H., Dunn A. J. (2007). Effects of interleukin-1*β* and lipopolysaccharide on behavior of mice in the elevated plus-maze and open field tests. *Pharmacology Biochemistry and Behavior*.

[16] Mullington J., Korth C., Hermann D. M. (2000). Dose-dependent effects of endotoxin on human sleep. *The American Journal of Physiology—Regulatory Integrative and Comparative Physiology*.

[17] Szentirmai É., Krueger J. M. (2014). Sickness behaviour after lipopolysaccharide treatment in ghrelin deficient mice. *Brain, Behavior, and Immunity*.

[18] Lawson M. A., Parrott J. M., McCusker R. H., Dantzer R., Kelley K. W., O'Connor J. C. (2013). Intracerebroventricular administration of lipopolysaccharide induces indoleamine-2,3-dioxygenase-dependent depression-like behaviors. *Journal of Neuroinflammation*.

[19] Azizi-Malekabadi H., Hosseini M., Soukhtanloo M., Sadeghian R., Fereidoni M., Khodabandehloo F. (2012). Different effects of scopolamine on learning, memory, and nitric oxide metabolite levels in hippocampal tissues of ovariectomized and sham-operated rats. *Arquivos de Neuro-Psiquiatria*.

[20] Wagner A. K., Willard L. A., Kline A. E. (2004). Evaluation of estrous cycle stage and gender on behavioral outcome after experimental traumatic brain injury. *Brain Research*.

[21] Pourganji M., Hosseini M., Soukhtanloo M., Zabihi H., Hadjzadeh M. A.-R. (2014). Protective role of endogenous ovarian hormones against learning and memory impairments and brain tissues oxidative damage induced by lipopolysaccharide. *Iranian Red Crescent Medical Journal*.

[22] Neamati A., Chaman F., Hosseini M., Boskabady M. H. (2014). The effects of *Valeriana officinalis* L. hydro-alcoholic extract on depression like behavior in ovalbumin sensitized rats. *Journal of Pharmacy and Bioallied Sciences*.

[23] Hosseini M., Zakeri S., Khoshdast S. (2012). The effects of *Nigella sativa* hydro-alcoholic extract and thymoquinone on lipopolysaccharide—induced depression like behavior in rats. *Journal of Pharmacy and Bioallied Sciences*.

[24] Boguszewski P., Zagrodzka J. (2002). Emotional changes related to age in rats—a behavioral analysis. *Behavioural Brain Research*.

[25] Cohen A., Whitfield T. W., Kreifeldt M. (2014). Virus-mediated shRNA knockdown of prodynorphin in the rat nucleus accumbens attenuates depression-like behavior and cocaine locomotor sensitization. *PLoS ONE*.

[26] Berardi A., Trezza V., Palmery M., Trabace L., Cuomo V., Campolongo P. (2014). An updated animal model capturing both the cognitive and emotional features of post-traumatic stress disorder (PTSD). *Frontiers in Behavioral Neuroscience*.

[27] Quan N., Sundar S. K., Weiss J. M. (1994). Induction of interleukin-1 in various brain regions after peripheral and central injections of lipopolysaccharide. *Journal of Neuroimmunology*.

[28] Takao T., Culp S. G., de Souza E. B. (1993). Reciprocal modulation of interleukin-1*β* (IL-1*β*) and IL-1 receptors by lipopolysaccharide (endotoxin) treatment in the mouse brain-endocrine-immune axis. *Endocrinology*.

[29] Bluthé R.-M., Dantzer R., Kelley K. W. (1992). Effects of interleukin-1 receptor antagonist on the behavioral effects of lipopolysaccharide in rat. *Brain Research*.

[30] Kang A., Hao H., Zheng X. (2011). Peripheral anti-inflammatory effects explain the ginsenosides paradox between poor brain distribution and anti-depression efficacy. *Journal of Neuroinflammation*.

[31] Layé S., Gheusi G., Cremona S. (2000). Endogenous brain IL-1 mediates LPS-induced anorexia and hypothalamic cytokine expression. *American Journal of Physiology—Regulatory Integrative and Comparative Physiology*.

[32] Miller A. H. (2010). Depression and immunity: a role for T cells?. *Brain, Behavior, and Immunity*.

[33] Piser T. M. (2010). Linking the cytokine and neurocircuitry hypotheses of depression: a translational framework for discovery and development of novel anti-depressants. *Brain, Behavior, and Immunity*.

[34] Leonard B. E. (2001). The immune system, depression and the action of antidepressants. *Progress in Neuro-Psychopharmacology and Biological Psychiatry*.

[35] Chourbaji S., Urani A., Inta I. (2006). IL-6 knockout mice exhibit resistance to stress-induced development of depression-like behaviors. *Neurobiology of Disease*.

[36] Maier S. F., Watkins L. R. (1995). Intracerebroventricular interleukin-1 receptor antagonist blocks the enhancement of fear conditioning and interference with escape produced by inescapable shock. *Brain Research*.

[37] Dunn A. J. (2006). Effects of cytokines and infections on brain neurochemistry. *Clinical Neuroscience Research*.

[38] Gao H.-M., Jiang J., Wilson B., Zhang W., Hong J.-S., Liu B. (2002). Microglial activation-mediated delayed and progressive degeneration of rat nigral dopaminergic neurons: relevance to Parkinson's disease. *Journal of Neurochemistry*.

[39] Dunn A. J., Wang J., Ando T. (1999). Effects of cytokines on cerebral neurotransmission: comparison with the effects of stress. *Advances in Experimental Medicine and Biology*.

[40] Dunn A. J. (2000). Cytokine activation of the HPA axis. *Annals of the New York Academy of Sciences*.

[41] Hritcu L., Gorgan L. D. (2014). Intranigral lipopolysaccharide induced anxiety and depression by altered BDNF mRNA expression in rat hippocampus. *Progress in Neuro-Psychopharmacology and Biological Psychiatry*.

[42] Amandusson Å., Blomqvist A. (2013). Estrogenic influences in pain processing. *Frontiers in Neuroendocrinology*.

[43] Li L.-H., Wang Z.-C., Yu J., Zhang Y.-Q. (2014). Ovariectomy results in variable changes in nociception, mood and depression in adult female rats. *PLoS ONE*.

[44] Ter Horst G. J., Wichmann R., Gerrits M., Westenbroek C., Lin Y. (2009). Sex differences in stress responses: focus on ovarian hormones. *Physiology and Behavior*.

[45] Walf A. A., Paris J. J., Frye C. A. (2009). Chronic estradiol replacement to aged female rats reduces anxiety-like and depression-like behavior and enhances cognitive performance. *Psychoneuroendocrinology*.

[46] Fedotova J. (2012). Effects of stimulation and blockade of D_2_ receptor on depression-like behavior in ovariectomized female rats. *ISRN Pharmacology*.

[47] Patki G., Allam F. H., Atrooz F. (2013). Grape powder intake prevents ovariectomy-induced anxiety-like behavior, memory impairment and high blood pressure in female Wistar rats. *PLoS ONE*.

[48] Rachman I. M., Unnerstall J. R., Pfaff D. W., Cohen R. S. (1998). Estrogen alters behavior and forebrain c-fos expression in ovariectomized rats subjected to the forced swim test. *Proceedings of the National Academy of Sciences of the United States of America*.

[49] Rehavi M., Sepcuti H., Weizman A. (1987). Upregulation of imipramine binding and serotonin uptake by estradiol in female rat brain. *Brain Research*.

[50] Blum I., Vered Y., Lifshitz A. (1996). The effect of estrogen replacement therapy on plasma serotonin and catecholamines of postmenopausal women. *Israel Journal of Medical Sciences*.

[51] Dunn A. J. (1992). Endotoxin-induced activation of cerebral catecholamine and serotonin metabolism: comparison with interleukin-1. *The Journal of Pharmacology and Experimental Therapeutics*.

[52] Disshon K. A., Dluzen D. E. (1997). Estrogen as a neuromodulator of MPTP-induced neurotoxicity: effects upon striatal dopamine release. *Brain Research*.

[53] Dluzen D. E., McDermott J. L., Liu B. (1996). Estrogen alters MPTP-induced neurotoxicity in female mice: effects on striatal dopamine concentrations and release. *Journal of Neurochemistry*.

[54] Disshon K. A., Boja J. W., Dluzen D. E. (1998). Inhibition of striatal dopamine transporter activity by 17beta-estradiol. *European Journal of Pharmacology*.

[55] Ho Y.-J., Tai S.-Y., Pawlak C. R., Wang A.-L., Cheng C.-W., Hsieh M.-H. (2012). Behavioral and IL-2 responses to diosgenin in ovariectomized rats. *The Chinese Journal of Physiology*.

[56] Pearce J., Hawton K., Blake F. (1995). Psychological and sexual symptoms associated with the menopause and the effects of hormone replacement therapy. *British Journal of Psychiatry*.

[57] Bassi G. S., Kanashiro A., Santin F. M., de Souza G. E. P., Nobre M. J., Coimbra N. C. (2012). Lipopolysaccharide-induced sickness behaviour evaluated in different models of anxiety and innate fear in rats. *Basic and Clinical Pharmacology and Toxicology*.

[58] Cruz A. P. M., Frei F., Graeff F. G. (1994). Ethopharmacological analysis of rat behavior on the elevated plus-maze. *Pharmacology Biochemistry and Behavior*.

[59] Rodgers R. J., Johnson N. J. T. (1995). Factor analysis of spatiotemporal and ethological measures in the murine elevated plus-maze test of anxiety. *Pharmacology Biochemistry and Behavior*.

[60] Andreatini R., Bacellar L. F. S. (1999). The relationship between anxiety and depression in animal models: a study using the forced swimming test and elevated plus-maze. *Brazilian Journal of Medical and Biological Research*.

[61] File S. E. (1993). The interplay of learning and anxiety in the elevated plus-maze. *Behavioural Brain Research*.

[62] Bertoglio L. J., Carobrez A. P. (2002). Prior maze experience required to alter midazolam effects in rats submitted to the elevated plus-maze. *Pharmacology Biochemistry and Behavior*.

[63] File S. E., Mabbutt P. S., Hitchcott P. K. (1990). Characterisation of the phenomenon of ‘one-trial tolerance’ to the anxiolytic effect of chlordiazepoxide in the elevated plus-maze. *Psychopharmacology*.

[64] Albrechet-Souza L., Oliveira A. R., de Luca M. C. Z., Tomazini F. M., Santos N. R., Brandão M. L. (2005). A comparative study with two types of elevated plus-maze (transparent vs. opaque walls) on the anxiolytic effects of midazolam, one-trial tolerance and fear-induced analgesia. *Progress in Neuro-Psychopharmacology and Biological Psychiatry*.

[65] Duncan G. E., Knapp D. J., Breese G. R. (1996). Neuroanatomical characterization of Fos induction in rat behavioral models of anxiety. *Brain Research*.

[66] Graeff F. G., Silveira M. C. L., Nogueira R. L., Audi E. A., Oliveira R. M. W. (1993). Role of the amygdala and periaqueductal gray in anxiety and panic. *Behavioural Brain Research*.

[67] Hinks G. L., Brown P., Field M., Poat J. A., Hughes J. (1996). The anxiolytics CI-988 and chlordiazepoxide fail to reduce immediate early gene mRNA stimulation following exposure to the rat elevated X-maze. *European Journal of Pharmacology*.

[68] Stone E. A., Zhang Y. (1995). Adrenoceptor antagonists block c-fos response to stress in the mouse brain. *Brain Research*.

[69] Mohammad G., Chowdhury I., Fujioka T., Nakamura S. (2000). Induction and adaptation of Fos expression in the rat brain by two types of acute restraint stress. *Brain Research Bulletin*.

[70] Beck C. H. M., Fibiger H. C. (1995). Conditioned fear-induced changes in behavior and in the expression of the immediate early gene c-fos: with and without diazepam pretreatment. *Journal of Neuroscience*.

